# The impact of characters like Tony the Tiger and other child-targeted techniques used in food and beverage marketing

**DOI:** 10.3389/fnut.2023.1287473

**Published:** 2023-12-05

**Authors:** Christine Mulligan, Lauren Remedios, Tim Ramsay, Elise Pauzé, Mariangela Bagnato, Monique Potvin Kent

**Affiliations:** ^1^School of Epidemiology and Public Health, Faculty of Medicine, University of Ottawa, Ottawa, ON, Canada; ^2^Interdisciplinary School of Health Sciences, Faculty of Health Sciences, University of Ottawa, Ottawa, ON, Canada

**Keywords:** food marketing, marketing power, children, cartoon characters, spokes-characters, child-targeted marketing, food policy

## Abstract

**Introduction:**

Food marketing’s impact is a function of exposure and power, both of which contribute to children’s poor diet quality and obesity risk. Children’s exposure to food marketing is well documented, however, few studies have assessed the impact of specific persuasive marketing techniques or aspects of ‘power’ on children.

**Methods:**

This study administered an online survey to 1,341 Canadian children (9–12 years) aiming to determine the impact of: (1) child-targeted vs. adult-targeted marketing, and (2) licensed characters vs. spokes characters on children’s food preferences and behavioral intentions. Participants were randomized to a single condition in each survey part and viewed 3 static food advertisements displaying the features of that condition (e.g., child-targeted advertising or licensed characters), and answered 3 Likert-scale (5-point) questions after each exposure. For each condition within each research question, there were four outcome variables related to the impact of marketing on children: food preference, purchase intent, pester power, and total impact. ANOVA tested the difference in impact (Likert scores) between conditions overall and for each outcome, with Bonferroni *post-hoc* tests where necessary.

**Results:**

A greater average total impact was observed among children exposed to child-targeted ads (mean Likert score 3.36) vs. adult-targeted ads (mean score 2.75; *p* < 0.001) or no marketing (mean score 2.81; *p* < 0.001). Children exposed to ads featuring spokes characters had a higher average total impact (mean score 3.98) vs. licensed characters (mean score 3.80; *p* < 0.001) and the control (i.e., no characters) (mean score 3.19; *p* < 0.001), and the total impact of licensed characters was greater than that of no characters. Similar trends were observed for all other outcomes.

**Discussion:**

Overall, this study showed that child-targeted ads and those using characters - especially spokes characters - have a strong overall impact on children’s food preferences, purchase intents, and pester power, and support the implementation of comprehensive marketing restrictions to protect children.

## 1 Introduction

The burden of childhood overweight, obesity and non-communicable diseases (NCDs) remains high globally, and in Canada (1–6). There is a well-established link between diet and nutrition-related chronic diseases such as obesity and in Canada, dietary risk is the top behavioral risk factor for death and disability following tobacco (7–11). Canadian children’s diets are consistently found to fall short of meeting national dietary guidelines; research shows that child diets are high in ultra-processed foods and low in fruits and vegetables putting them at risk for nutrition-related chronic disease (12–15).

Food marketing has been highlighted as an important causal factor contributing to poor diet quality in children, and to childhood obesity (16–20). Canadian children are exposed to a high volume of food marketing across various media platforms and settings, including television, digital and social media, at school, and in recreational centers, among others (21–26). Recent data from Canada has shown that there were 54 million food and beverage ads on the top 10 child-preferred websites alone over a one-year period, and that children aged 2–11 years in Toronto were exposed to 2,234 food ads in 2019 on television across 36 stations (27). This exposure was propelled by the estimated 628 million dollars in food and beverage advertising expenditures that occurred in Canada in 2019, most of which occurred on television (68%) and digital media (12%) (28). There is also a plethora of evidence indicating that the vast majority of marketing children are exposed to promotes food and beverage products that are of poor nutritional quality that are often high in sodium, sugars and fat (18, 29, 30). Children are particularly vulnerable to the effects of marketing and a series of systematic reviews have documented that unhealthy food marketing impacts children’s food preferences, intakes, and requests (16, 18–20). As a result, the World Health Organization (WHO) has recommended that countries develop policies to restrict these marketing practices (30, 31).

The overall impact of food marketing is a function of both children’s exposure to food marketing, and the power of such marketing (31). While “exposure” refers to the reach, and frequency of the marketing, “power” refers to its content and design (31). While the bulk of the scientific literature has focused on child exposure to food marketing, research has also documented the power of food and beverage marketing. Although, the types of techniques that are used varies between media (e.g., print media vs. digital), there are many techniques that are consistent across all marketing platforms, such as the use of: promotional characters or brand spokes-characters (like Tony the Tiger), nutrition or health appeals, taste appeals, celebrity endorsements, colorful or eye-catching visual imagery, appeals to fun or humor, emotional appeals, child-appealing themes (e.g., fantasy, adventure), games, toys, giveaways, contests, and more (18, 32–35). Research from Canada studying the power of marketing has elucidated similar trends in the types of strategies manufacturers are employing to appeal to children (25, 26, 36–40).

While there is a growing body of literature describing the power of food marketing, fewer studies have assessed the impact of specific persuasive marketing techniques or aspects of ‘power’ on children. The use of advergames, for example, have been found to impact children’s food choice and intakes (41–44). While some studies have examined and highlighted the impact of various characters on children’s attention, recall, preferences, and choice of products (16, 41, 45–53), there are many gaps regarding the impact of specific techniques compared to others. For instance, despite characters being a frequently displayed and generally impactful marketing technique, it is unknown how various types of characters, such as brand spokes characters or licensed characters (i.e., from popular movies or television shows) differentially impact children.

There has also been recent research indicating that children are drawn to marketing techniques that are not typically considered to be targeted at youth, such as appeals to health and nutrition or giveaways and promotions for adult-targeted products (e.g., prepaid gas cards) (54). This is important as children are also heavily exposed to food and beverage marketing targeting older demographics, within child-focused media or settings (e.g., adult-targeted ad featured on a children’s television channel), while frequenting mixed-audience settings (e.g., professional sports games) or while consuming mixed-audience media (e.g., prime time television). To date, however, there have been no studies to our knowledge which have specifically studied the impact of adult-targeted food marketing (i.e., with the absence of marketing techniques specifically targeting children) on children’s preferences or made comparisons to child-targeted marketing.

Assessing these nuances in impact between different aspects of marketing power is essential to understanding how the specific content and features of food and beverage marketing play a role in children’s food preferences and food-related behaviors. These questions have yet to be investigated in the Canadian context and such evidence is critical to informing the development of comprehensive marketing policies that are in line with WHO guidance and ensuring all types of marketing that impact children are being restricted (30, 55, 56). As such, this study aimed to answer two research questions: (1) what is the impact of adult-targeted food and beverage advertisements compared to child-targeted food beverage advertisements on children’s food preferences and behavioral intentions; and (2) What is the impact of spokes-characters vs. licensed characters used in food and beverage advertisements on children’s food preferences and behavioral intentions? The authors hypothesize that child-targeted advertisements will have a stronger impact on children than adult-targeted advertisements, and that there will be no difference in impact between advertisements featuring spokes-characters and licensed characters.

## 2 Materials and methods

This study was a cross-sectional study; an online survey was administered to over 1,000 Canadian children to determine the impact of (1) child-targeted vs. adult-targeted food and beverage ads, and (2) ads featuring licensed characters vs. spokes characters. This study was approved by the University of Ottawa Research Ethics Board (H-11-22-8517).

### 2.1 Participants and recruitment

Participants were recruited for this study by the market research company, Leger. Leger targeted (via email) adult panelists who identify as being parents of children within the intended study demographic by email. For this study, recruitment was aimed at children aged 9–12 years old living in Canada, speaking English or French and having the ability to complete an online survey. Parents were asked a series of screening questions to determine eligibility and those who met the inclusion criteria were asked to provide informed consent for their child to participate in the survey; children also provided informed assent. Participants were able to complete the survey either in English or in French. Participants were compensated per Leger’s usual incentive structure.

Given the study design required to answer the research questions, we aimed to recruit 1,000 children for this study. Based on a recent systematic review and meta-analysis, we anticipated a small effect size (i.e., standard mean difference of 0.3) of food marketing on children’s preferences (16). To detect a significant difference of that magnitude between 2 groups in a 2-tailed *T* test with 80% power, the minimum sample size required per study group was 175 participants. With a sample size of 1,000 children, all conditions for all parts of the study would have at minimum 250 participants, providing adequate power for any given comparison. This number of participants also aligned with budgetary limitations and recruiting feasibility as assessed by Leger. Recruitment was conducted as to be nationally representative (based on provincial population), and quota sampling was used to obtain equal numbers of males/females and children aged 9–10 and 11–12 years. Participants were compensated according to Leger’s usual incentive structure. In total, *n* = 1,341 children completed the survey administered by Leger.

### 2.2 Experimental design

To test the research questions, a survey was administered online to participants by Leger. The first part of the survey consisted of a short demographic questionnaire that was completed by parents on behalf of their child, which asked questions about the child’s age, sex, ethnicity, and perceived income adequacy. Children then completed the remainder of the survey on their own.

The children’s portion of the survey consisted of 2 parts, each corresponding to one of the research questions on the impact of food and beverage ads: (1) child-targeted vs. adult-targeted (RQ1), and (2) licensed characters vs. spokes characters (RQ2). A summary of the survey parts and conditions is presented in Table 1. Participants were randomized to a single condition within each part of the survey, for which they were asked to view 3 static food advertisements (in random order) displaying the features of that condition (e.g., child-targeted advertising or licensed characters). The order of the survey parts was also randomized. In total, children viewed and responded to 6 advertisements over the course of the whole survey. It is important to note that the present study was conducted as part of a larger study on the impact of food marketing on children. There were thus additional survey parts that were administered to participants to address other research questions, however, only those relevant to the current research will be discussed here.

**Table 1:** Summary of survey parts and conditions.

	Part 1: RQ 1	Part 2: RQ 2
Conditions	Child-targeted ad	Licensed characters
	Adult-targeted ad	Spokes characters
	No marketing (control)	No characters (control)

**Figure 1:**
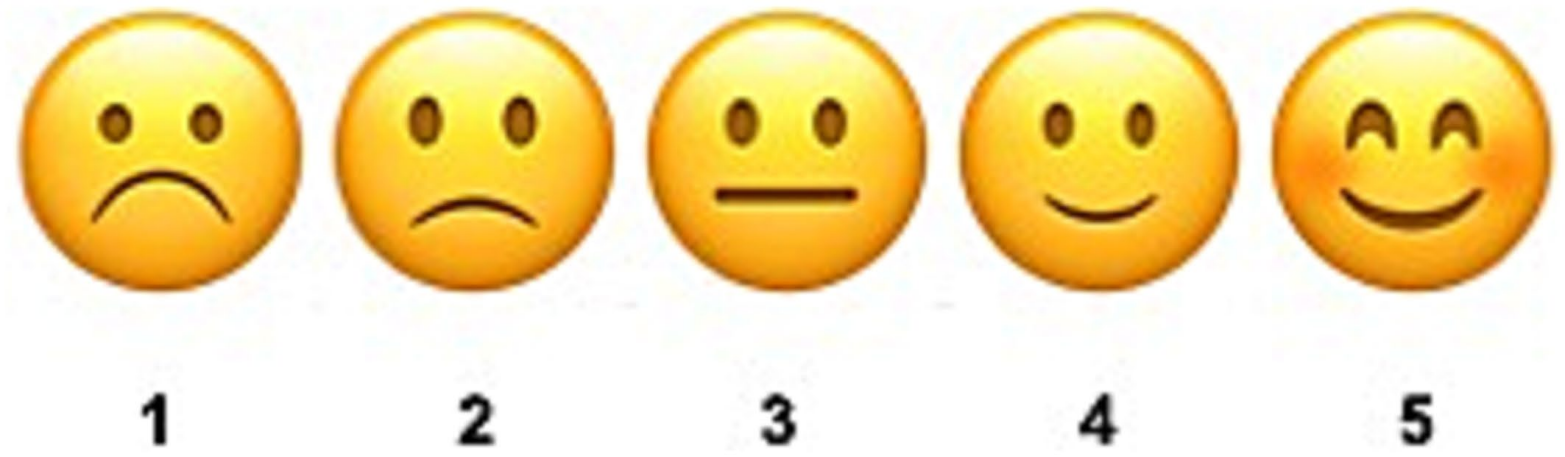
Likert scale scores and emojis.

Following each ad exposure, participants were asked to answer the following Likert-scale questions (5-points, indicated by emojis ranging from sad (1) to happy (5) faces; Figure 1) related to their preference, purchase intent and pester power, respectively: (1) How much would you like to eat/drink this product; (2) Would you choose to buy this product in a store, and (3) Would you ask an adult to buy this product for you?

The ad images children were exposed to were designed specifically for this study. The 3 ads within an individual condition were designed to display similar features relevant to that condition, but differed in terms of the product shown and the specific design of the ad. For instance, within the “child-targeted ads” condition, all images would display a variety of child-targeted marketing techniques (e.g., bright colors, fun themes, child language, cartoons, etc.), but would feature different food products (e.g., yogurt, cereal or granola bars). Similarly, in the “licensed character” and “spokes-character” conditions, different characters (of the same type) were featured in each of the 3 images presented in both conditions. All ads were designed to be gender-neutral (e.g., avoiding stereotypical gendered advertising techniques or characters such as princesses or race cars) and appropriate for children within the study age range. Where possible, ads were for products from brands unfamiliar to children in Canada (i.e., brands from the United Kingdom or Australia) to reduce bias due to pre-existing brand or product preferences. In some cases (e.g., RQ2 – spokes character condition), this was not feasible given the nature of the condition. Additionally, where possible, products featured in the ad images were from “health-neutral” food categories (i.e., not ‘junk foods’, e.g., yogurt, cereal, granola bars) to avoid bias based on children’s known preference for junk-foods (57).

### 2.3 Outcomes and analysis

Demographic variables were analyzed descriptively. For each condition within each research question, there were four outcome variables of interest related to the impact of marketing on children: (1) Food preference (score from Likert question 1); (2) Purchase intent (Likert question 2); (3) Pester power (Likert question 3); (4) Total impact (average of all Likert scores). These outcome variables are key components of the commonly referenced hierarchy of unhealthy food promotion effects on children proposed by Kelly et al. (58). These outcomes have also been highlighted in most recent WHO-commissioned systematic review and meta-analysis on the impacts of food marketing on children (16). In this survey, a Likert score of 3 was represented by a “neutral face” emoji, so for the purposes of these analyses, an average Likert score greater than 3 (i.e., happy faces) can be interpreted as a positive impact on children, and any score lower than 3 (i.e., sad faces) can be interpreted as a negative impact.

To evaluate the difference in impact between each condition on preference, purchase intent, pester power, and total impact, for each RQ analysis of variance (ANOVA) models were fitted with Likert scores for food preference, purchase intent, pester power and total impact as outcomes; sex (male/female), age (9–10 years/11–12 years), ethnicity (majority, minority), perceived income adequacy (low/high), and condition as fixed factors/independent variables. There was no interaction between condition, age, and sex, so further subgroup analyses were not conducted. In cases where the ANOVA yielded significant results, Bonferroni post-hoc tests were conducted. Results were considered statistically significant when *p* < 0.05. All data was analyzed using Microsoft Excel and SPSS 27.0 (IBM, 2020).

## 3 Results

Sociodemographic characteristics of the participants (*n* = 1,341) are presented in Table 2. A total of 49.2% of the sample was male and 50.6% was female and the average age of participants was 10.6 years (47.4% 9–10 years and 52.6% 11–12 years). Most participants identified as being in the ethnic majority group (i.e., White, 64.5%) and high perceived income adequacy (60%). An expanded summary of all collected sociodemographic data can be found in Supplementary Table S1.

**Table 2:** Demographic characteristics of the study sample (*n* = 1,341).

	*n*	% of total
Total sample	1,341	100.0
Sex		
Female	679	50.6
Male	660	49.2
Prefer not to say	2	0.1
Age		
11–12 years	706	52.6
9–10 years	635	47.4
Mean Age (SD)	10.6 (1.1) years	
Ethnicity^1^		
Majority	869	64.8
Minority	457	34.1
Did not answer	15	1.1
Perceived income adequacy^2^		
High	804	60.0
Low	530	39.5
Did not answer	7	0.5
Province/Region of residence		
West (British Columbia, Alberta)	323	22.6
Prairies (Saskatchewan, Manitoba)	91	6.4
Ontario	523	36.5
Quebec	318	22.2
East (Newfoundland and Labrador, Nova Scotia, New Brunswisk, Prince Edward Island)	85	5.9
North (Yukon, Northwest Territories, Nunavut)	1	0.1

^1^Ethnicity was categorized as “majority” (i.e., only “White (European descent)” was selected) and “minority” (i.e., any other ethnicity group(s) were selected, including when in addition to “White (European descent)” being selected).

^2^Perceived income adequacy was categorized as “high” (Reponses of either very easy, easy, and neither easy nor difficult when asked how difficult or easy it is for you to make ends meet?) or “low” (responses of difficult or very difficult).

### 3.1 RQ1: child-targeted vs. adult-targeted ads

**Figure 2:**
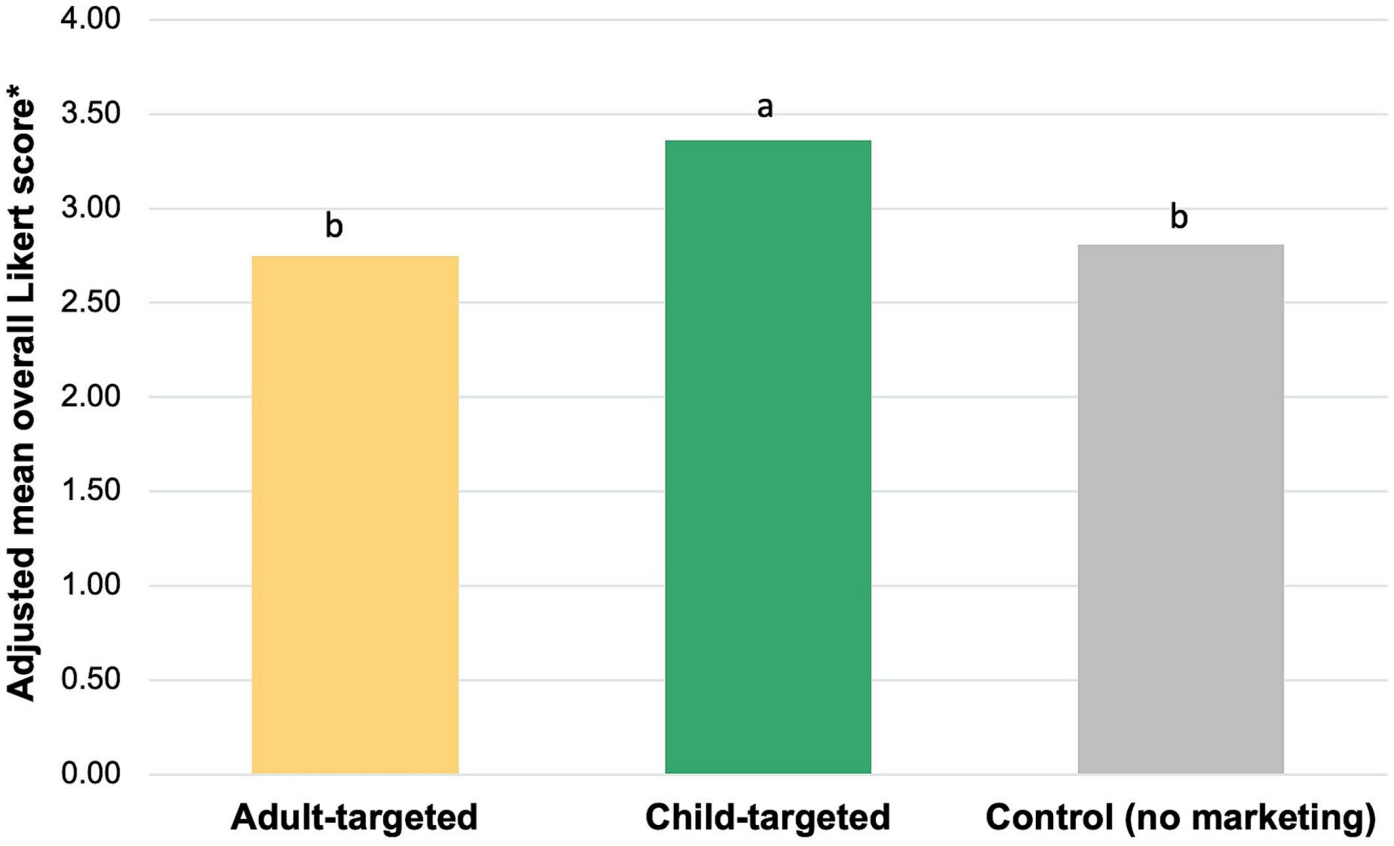
Total impact of child-targeted vs. adult targeted ads on children’s food preferences and behavioral intentions. Bars that do not share subscripts have means that differ by *p* < 0.05 according to Bonferroni multiple comparisons.

The effects of exposure to adult vs. child-targeted ads, and exposure to child-targeted ads vs. the control (no marketing) on total impact, were significantly different (Figure 2). A significantly greater average total impact was observed among children exposed to child-targeted ads (mean Likert score 3.36) compared to those exposed to adult-targeted ads (mean score 2.75; *p* < 0.001) or no marketing (mean score 2.81; *p* < 0.001).

As shown in Table 3, average preference, purchase intent, and pester responses differed significantly by ad exposure condition, overall and by ethnicity. Average food preference was significantly higher among participants exposed to child-targeted ads (mean score 3.38) compared to both adult-targeted ads (mean score 2.83; *p* < 0.001) or control (mean score 2.87; *p* < 0.001). Similarly, average purchase intent and pester power responses were also significantly higher among those exposed to child-targeted ads (mean scores 3.33 and 3.38, respectively) compared to adult (2.72 and 2.70; *p* < 0.001) or control conditions (2.79 and 2.78; *p* < 0.001). Among ethnic minorities and majorities, preference, purchase, and pester were significantly higher among those exposed to child-targeted ads compared to those exposed to adult-targeted or no marketing (control), with majority ethnicity participants reporting stronger impact. There was no significant interaction effect between sex, age, perceived income adequacy, and ad exposure condition on preference, purchase, pester, or total impact responses.

**Table 3:** Total impact and impact of child-targeted vs. adult-targeted ads on children’s food preference, purchase intent and pester power.

Condition	Adult-targeted marketing	Child-targeted marketing	Control (no marketing)	
Food preference				
	Adjusted mean^1^	Adjusted mean^1^	Adjusted mean^1^	*p* value^2^
Overall	2.83^b^	3.38^a^	2.87^b^	*p* < 0.01
Sex				0.57
Male	2.84	3.35	2.91	
Female	2.83	3.41	2.83	
Age				0.74
9–10 years	2.9	3.44	2.89	
11–12 years	2.77	3.33	2.85	
Ethnicity^3^				0.02
Minority	2.85^b^	3.30^a^	2.97^b^	
Majority	2.82^b^	3.46^a^	2.77^b^	
Perceived income adequacy^4^				0.27
Low	2.82	3.4	2.79	
High	2.84	3.36	2.96	
Purchase intent				
	Adjusted mean	Adjusted mean	Adjusted mean	*p* value
Overall	2.72^b^	3.33^a^	2.79^b^	*p* < 0.01
Sex				0.67
Male	2.74	3.31	2.83	
Female	2.71	3.35	2.76	
Age				0.66
9–10 years	2.79	3.38	2.8	
11–12 years	2.66	3.28	2.79	
Ethnicity				0.01
Minority	2.76^b^	3.26^a^	2.92^b^	
Majority	2.69^b^	3.40^a^	2.67^b^	
Perceived income adequacy				0.09
Low	2.73	3.36	2.68	
High	2.72	3.3	2.9	
Pester power				
	Adjusted mean	Adjusted mean	Adjusted mean	*p* value
Overall	2.70^b^	3.38^a^	2.78^b^	*p* < 0.01
Sex				0.51
Male	2.71	3.34	2.82	
Female	2.69	3.42	2.74	
Age				0.29
9–10 years	2.77	3.44	2.75	
11–12 years	2.63	3.32	2.81	
Ethnicity				0.03
Minority	2.73^b^	3.33^a^	2.91^b^	
Majority	2.67^b^	3.42^a^	2.64^b^	
Perceived income adequacy				0.21
Low	2.69	3.4	2.68	
High	2.71	3.36	2.88	
Total impact				
	Adjusted mean	Adjusted mean	Adjusted mean	*p* value
Overall	2.75^b^	3.36^a^	2.81^b^	*p* < 0.01
Sex				0.56
Male	2.76	3.33	2.85	
Female	2.74	3.4	2.78	
Age				0.52
9–10 years	2.82	3.42	2.81	
11–12 years	2.68	3.31	2.82	
Ethnicity				0.02
Minority	2.78^b^	3.30^a^	2.94^b^	
Majority	2.73^b^	3.43^a^	2.69^b^	
Perceived income adequacy				0.16
Low	2.75	3.39	2.72	
High	2.76	3.34	2.91	

^1^Adjusted means based on ANOVA models fitted with Likert scores for food preference, purchase intent, pester power and total impact as outcomes; sex (male/female), age (9–10 years/11–12 years), ethnicity (majority, minority), perceived income adequacy (low/high), and condition as fixed factors/independent variables.

^2^*p* values <0.05 were considered to be statistically significant, with differences between conditions indicated by differing superscript letters.

^3^Ethnicity was categorized as “majority” (i.e., only “White (European descent)” was selected) and “minority” (i.e., any other ethnicity group(s) were selected, including when in addition to “White (European descent)” being selected).

^4^Perceived income adequacy was categorized as “high” (Reponses of either very easy, easy, and neither easy nor difficult when asked how difficult or easy it is for you to make ends meet?) or “low” (responses of difficult or very difficult).^a,b^Means that do not share subscripts have means that differ by *p* < 0.05 according to Bonferroni multiple comparisons.

### 3.2 RQ2: licensed characters vs. spokes characters

**Figure 3:**
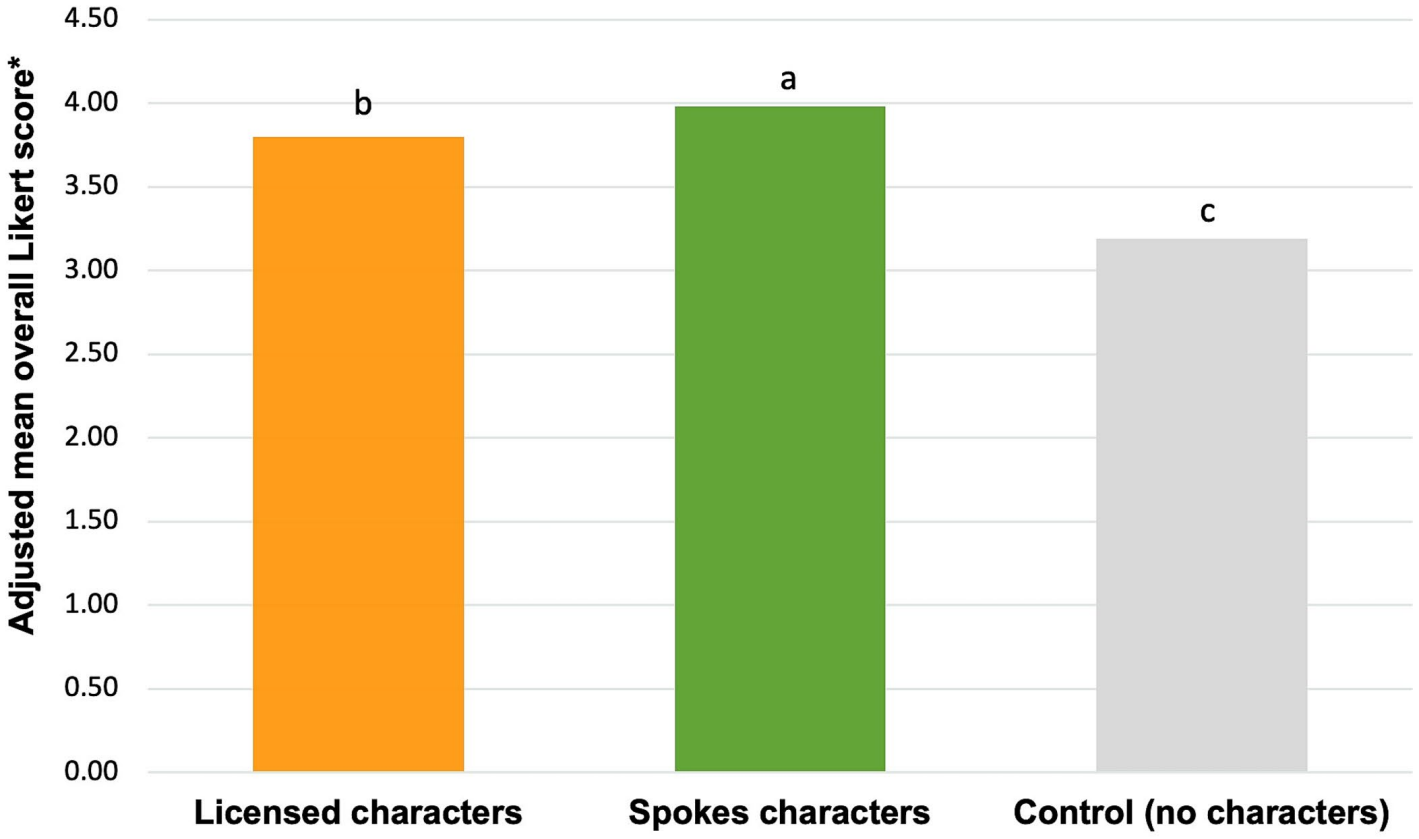
Total impact of licensed characters vs. spokes characters on children’s food preferences and behavioral intentions. Bars that do not share subscripts have means that differ by *p* < 0.05 according to Bonferroni multiple comparisons.

The average total impact significantly differed per condition (Figure 3). Children exposed to ads featuring spokes characters had a significantly higher average total impact (mean score 3.98) compared to those exposed to licensed characters (mean score 3.80; *p* < 0.001) and the control (i.e., no characters) (mean score 3.19; *p* < 0.001).

The effect of exposure to spokes characters on food preference (mean score 4.02), purchase (3.93), and pester power (4.00) responses was greater compared to those exposed to licensed characters (mean scores 3.84, 3.79, 3.78, respectively; *p* < 0.001) or no characters (3.25, 3.16, 3.17, respectively; *p* < 0.001) while exposure to licensed characters was significantly different to no characters (*p* < 0.001) (Table 4). The response outcomes did not significantly differ by the interaction effect between ad exposure condition, sex, age, ethnicity, or perceived income adequacy.

**Table 4:** Total impact and impact of licensed characters vs. spokes characters on children’s food preference, purchase intent and pester power.

Condition	Licensed characters	Spokes characters	Control (no characters)	
Food preference				
	Adjusted mean^1^	Adjusted mean^1^	Adjusted mean^1^	*p* value^2^
Overall	3.84^b^	4.02^a^	3.25^c^	*p* < 0.001
Sex				0.22
Male	3.84	3.97	3.3	
Female	3.84	4.07	3.19	
Age				0.12
9–10 years	3.91	4.17	3.27	
11–12 years	3.77	3.87	3.22	
Ethnicity^3^				0.37
Minority	3.81	3.96	3.27	
Majority	3.87	4.08	3.22	
Perceived income adequacy^4^				0.11
Low	3.84	4.03	3.14	
High	3.84	4.02	3.35	
Purchase intent				
	Adjusted mean	Adjusted mean	Adjusted mean	*p* value
Overall	3.79^b^	3.93^a^	3.16^c^	*p* < 0.001
Sex				0.44
Male	3.78	3.89	3.2	
Female	3.81	3.98	3.12	
Age				0.3
9–10 years	3.87	4.07	3.19	
11–12 years	3.71	3.8	3.13	
Ethnicity				0.4
Minority	3.78	3.87	3.18	
Majority	3.8	4	3.14	
Perceived income adequacy				0.07
Low	3.84	3.97	3.07	
High	3.75	3.9	3.25	
Pester power				
	Adjusted mean	Adjusted mean	Adjusted mean	*p* value
Overall	3.78^b^	4.00^a^	3.17^c^	*p* < 0.001
Sex				0.54
Male	3.75	4.01	3.21	
Female	3.81	3.99	3.13	
Age				0.43
9–10 years	3.84	4.13	3.23	
11–12 years	3.72	3.87	3.12	
Ethnicity				0.18
Minority	3.75	3.94	3.23	
Majority	3.82	4.06	3.12	
Perceived income adequacy				0.2
Low	3.84	4.02	3.12	
High	3.72	3.98	3.23	
Total impact				
	Adjusted mean	Adjusted mean	Adjusted mean	*p* value
Overall	3.80^b^	3.98^a^	3.19^c^	*p* < 0.001
Sex				0.44
Male	3.79	3.96	3.24	
Female	3.82	4.01	3.15	
Age				0.24
9–10 years	3.87	4.12	3.23	
11–12 years	3.74	3.84	3.16	
Ethnicity				0.28
Minority	3.78	3.92	3.23	
Majority	3.83	4.05	3.16	
Perceived income adequacy				0.11
Low	3.84	4.01	3.11	
High	3.77	3.96	3.28	

^1^Adjusted means based on ANOVA models fitted with Likert scores for food preference, purchase intent, pester power and total impact as outcomes; sex (male/female), age (9–10 years/11–12 years), ethnicity (majority, minority), perceived income adequacy (low/high), and condition as fixed factors/independent variables.

^2^*p* values <0.05 were considered to be statistically significant, with differences between conditions indicated by differing superscript letters.

^3^Ethnicity was categorized as “majority” (i.e., only “White (European descent)” was selected) and “minority” (i.e., any other ethnicity group(s) were selected, including when in addition to “White (European descent)” being selected).

^4^Perceived income adequacy was categorized as “high” (Reponses of either very easy, easy, and neither easy nor difficult when asked how difficult or easy it is for you to make ends meet?) or “low” (responses of difficult or very difficult).^a–c^Means that do not share subscripts have means that differ by *p* < 0.05 according to Bonferroni multiple comparisons.

## 4 Discussion

The overarching objective of this study was to determine how various aspects of marketing power (i.e., the design, content, and overall impression) impact children’s food preferences and behavioral intentions. Two research questions examined the differential impact of child-targeted vs. adult-targeted ads, and licensed characters vs. spokes characters.

This study found that child-targeted ads had a positive impact on children’s preferences, purchase intents, pester power and total impact. These results differed significantly from the impact of adult-targeted ads, and ads with no marketing (control condition), both of which had negative impacts on all examined outcomes. This indicates that when children are exposed to food and beverage marketing, the ads that display features of child-targeted marketing are most likely to trigger children’s desire to consume, purchase or pester parents about those products, especially in comparison to ads targeting adults or those with little to no marketing power. These findings are supported by previous literature on the impact of child-targeted food and beverage marketing on children’s food preferences and food-related behaviors (16, 19, 20, 59, 60). For instance, the most recent systematic review and meta-analysis on this topic reported that exposure to food marketing was associated with increases in children’s food intakes, food choices and purchase requests of marketed products (16). However, this review did not delineate between the impact of marketing that employed child-targeted techniques and marketing that did not; the present study contributes evidence to fill this gap. Our results are concerning, when considered in conjunction with the evidence speaking to the volume of child-targeted marketing Canadian children are exposed to and the consistently poor nutritional quality of the products being promoted by this marketing (18, 29, 30). Advertisements featuring powerful, child-targeted marketing techniques are likely increasing children’s desire to consume, purchase and pester for products that will negatively impact their diet quality and health outcomes, and must be restricted.

The adult-targeted ads had a slightly negative impact on children in our study and this result is discordant with other studies that have spoken to the appeal of marketing techniques that are not explicitly child-targeted or that are aimed at older demographics (54). However, this research question was aiming to evaluate the overall impression of the ad, rather than the specific marketing techniques that were used, meaning that while, overall, adult-targeted ads were less impactful on children in our study, it is still possible that specific adult-targeted marketing techniques are appealing to children. It is worth noting that to date, there have still been few studies aiming to elucidate the impacts of marketing techniques beyond those implicitly targeting children, and further research should aim to determine which adult-targeted techniques (such as health claims and giveaways or price promotions targeting adults) are most impactful to children, or how the use of these techniques in conjunction with child-targeted marketing techniques influences the overall impact of the marketing on children.

Some literature has noted potential differences in marketing impact based on demographic characteristics (e.g., age, sex, gender, weight status, socioeconomic status) (18, 61–63). For instance, a study from the UK found that following exposure to food marketing, children with obesity or excess weight had larger increases in snack intake compared to children with normal weight status (61). A recent Canadian study found that older youth (aged 13–17) reported higher exposure to food marketing online, females reported higher marketing exposure online and in retail settings, while males were exposed more frequently in video games; and that youth from minority ethnic groups and households with lower income adequacy reported higher exposure to marketing (63). This study, however, did not assess the impact of this exposure on differing demographics. The present study found no effect of age, sex, or perceived income adequacy on marketing impact of child- or adult-targeted ads. This can likely be explained in part by the fact that the static ad images used in this experiment were designed to be gender -neutral and appealing to a broad age-range of children to reduce bias. In real world settings, however, children’s personal characteristics almost certainly play a role in the impact of the food marketing they see. One recent study has attempted to elucidate how characteristics of Canadian children (e.g., sociodemographic, behavioral, and dietary intake factors) impact the appeal of real-world instances of digital food marketing (64). The authors report that there was large variability in what children found appealing and that the power of marketing instances varied even within groups of children with similar characteristics, suggesting that children’s marketing preference may largely be personal and not linked to sociodemographic group membership (64). Interestingly, our results indicated that child-targeted ads had a stronger total impact and impact on preference, purchase intent and pester power in the majority (i.e., White) ethnic group. While there has been some recent evidence documenting potential inequities in marketing exposure, whereby children’s exposure to food and beverage marketing seems to be higher in lower socioeconomic status (SES) and racialized communities (18, 65), there is a paucity of evidence examining the impact of food marketing across sociodemographic strata especially in Canada, and further research is needed in this area to consolidate these findings and ensure that any future marketing policies are equitable.

The second research question addressed by this study delved into one specific child-targeted marketing technique: the display of characters. Results showed that spokes characters had the strongest total impact on children compared to licensed characters and the control condition. While not as strong of an impact, licensed characters still had a positive impact on children, which was significantly greater the impact of marketing that did not display any characters (control condition). In line with previous literature speaking to the powerful impact of characters (16, 41, 45–48), this study found that ads featuring spokes characters and licensed characters increased children’s desire to consume, purchase or pester parents about products in comparison to ads that did not feature these marketing techniques, with spokes characters being the most powerful of the two examined character types. Research has shown that children’s characters are one of the marketing techniques that children are most exposed to on many different media platforms and settings where children live and play (18, 32–35). Manufacturers are evidently choosing to employ this marketing technique frequently, likely because they have found it to be valuable for building brand equity and effective at increasing purchasing and therefore, profits. The ethics of using characters to promote foods and beverages to children has been questioned, and some have called for greater accountability from companies regarding their use of spokes and licensed characters in order to protect children’s health (66). However, given that major food and beverage companies have a fiduciary duty to their shareholders that conflict with prioritizing public health (e.g., generating profit), governments should take responsibility for ensuring children are not unduly exposed to harmful food and beverage marketing by introducing federally mandated policies.

Findings from RQ1 indicated that child-targeted marketing is impactful to children, and these results add nuance to these findings by highlighting a specific marketing technique that is contributing to the overall child-targeted impression of the ad and boosting its impact. Findings such as these, examining the impact of individual marketing techniques, are important, as they provide a strong rationale for including these aspects of power within marketing restrictions in order to most effectively protect children from the aspects of food marketing that are having the strongest impacts on them and consequently, their dietary health. As such, additional research should aim to examine the impact of other marketing techniques, especially emergent marketing techniques such as user-generated content on social media, which has been found to be increasingly prevalent in Canada (67). For instance, some research has focused on examining the impact of social media, influencers and advergaming, on children’s food-related behaviors, and have found this type of marketing to be incredibly powerful (68, 69). Data such as these should absolutely be considered by policymakers when aiming to develop effective marketing regulations. In terms of developing marketing policies, the WHO recommends a mandatory, comprehensive approach that restricts all forms of marketing to children of foods which are high in saturated fats, trans-fatty acids, free sugars, or salt (56). Their guidelines further indicate that along with reducing exposure, policies should also aim to reduce the power of food marketing. The results of this work support this guidance as characters and other elements of child-targeted marketing were found to impact children’s food behaviors. Importantly, this study highlights the need for broad definitions of what constitutes “child-targeted” marketing within the scope of marketing policies (i.e., including multiple aspects of marketing power), in order to ensure that children are adequately protected from the persuasive power of food marketing.

This study presented the first Canadian examination of the impact of (1) child-targeted vs. adult-targeted food and beverage marketing, (2) marketing featuring licensed characters vs. spokes characters on children’s food preference, purchase intent, and pester power, strengthened by the use of a large and nationally representative sample of Canadian children. Strong efforts were made to reduce bias from pre-existing preferences and brand attitudes, or random error, namely by using multiple ad exposures per condition, as well the intentional design of the survey ad images to be gender-neutral and display unfamiliar products/brands and health-neutral food categories when possible. Randomization was also employed in several ways. Study participants were randomly assigned to an ad exposure condition within each research question, and this helped to achieve a relatively equal distribution of participants within each condition based on sociodemographic variables (i.e., sex and age). Participants also viewed each ad exposure within their assigned condition in randomized order to further protect against bias. The order of which participants were exposed to each part of the survey (i.e., each RQ) was also random. Finally, the strengths of the analytical approach employed in this study, in particular the use of ANOVA analysis, allowed for results to be compared between conditions, while adjusting for relevant sociodemographic variables. Moreover, *post hoc* Bonferroni tests enabled the identification of significant pairwise comparisons and providing additional depth to the analysis. This study was, however, not without limitations, some inherent to survey study design, such as survey fatigue, which may have impacted the quality and accuracy of responses. Next, the study sample primarily consisted of participants identifying as ethnic majority and of higher income which may have reduced the generalizability of the results, however this is a skew is commonly observed when recruiting participants from online/online survey panels (70). Additionally, the effect of BMI or weight-status on the response outcomes could not be assessed in this study due to inconsistent or incomplete self-reporting of participants’ height and weight observed in this survey. Finally, it is necessary to acknowledge that children have individualized preferences (e.g., naturally prefer one character over another) and were only exposed to 3 images per condition for feasibility reasons and to limit participant fatigue. While the marketing images used in this study were designed with the intention of being as universally appealing as possible, it is plausible that this may have somewhat neutralized the overall impact of the ads on some children, or that the selected images did not capture the interest of some children at all. As well, our results may not be generalizable to all food advertising (e.g., other food categories), and other unmeasured factors may explain differences in responses to different types of advertising conditions (e.g., familiarity of characters). In an expanded study or a real-world setting, greater variability or strength in the response outcomes could be expected, especially on an individual level, given that children are exposed to a large volume and variety of marketing on a daily basis that may better align with their personal preferences and therefore increase its impact.

This study showed that child-targeted ads and those using characters - especially spokes characters - have a strong overall impact on children’s food preferences, purchase intents, and pester power. Taken together, the results of this research provide timely evidence to support and inform the development and implementation of federally mandated marketing restrictions in Canada and highlight the importance of carefully considering aspects of marketing power within the regulatory approach to best protect children from the harmful effects of food marketing.

## Data availability statement

The raw data supporting the conclusions of this article will be made available by the authors, without undue reservation.

## Ethics statement

The studies involving humans were approved by University of Ottawa Research Ethics Board. The studies were conducted in accordance with the local legislation and institutional requirements. Written informed consent for participation in this study was provided by the participants’ legal guardians/next of kin.

## Author contributions

CM: Conceptualization, Formal analysis, Investigation, Methodology, Visualization, Writing – original draft, Writing – review & editing. LR: Conceptualization, Formal analysis, Investigation, Methodology, Writing – review & editing. TR: Conceptualization, Formal analysis, Writing – review & editing. EP: Conceptualization, Writing – review & editing. MB: Conceptualization, Writing – review & editing. MP: Conceptualization, Funding acquisition, Supervision, Writing – review & editing.
