# Supplementary material for: The impact of characters like Tony the Tiger and other child-targeted techniques used in food and beverage marketing
Source: Front Nutr. 2023 Dec 5;10:1287473. doi: 10.3389/fnut.2023.1287473 (PMC10728630; doi:10.3389/fnut.2023.1287473)
Supplement: Supplementary file 1 [file Table_1.DOCX]

Supplementary Material

# Supplementary Tables

**Supplementary Table 1. Expanded summary of self-reported sociodemographic data collected from study participants.**

|  | **n** | % |
| --- | --- | --- |
| Total Sample | 1341 | 100.0 |
| Sex | | |
| Female | 679 | 50.6 |
| Male | 660 | 49.2 |
| Prefer not to say | 2 | 0.1 |
| Age | | |
| 11-12 years | 706 | 52.6 |
| 9-10 years | 635 | 47.4 |
| Mean (SD) | 10.6 (1.1) |  |
| Ethnicity^1^ | | |
| Majority | 869 | 64.8 |
| Minority | 457 | 34.1 |
| Prefer not to say | 15 | 1.1 |
| Perceived Income Adequacy^2^ |  |  |
| High | 804 | 60.0 |
| Low | 530 | 39.5 |
| Prefer not to say | 7 | 0.5 |
| Province of Residence | | |
| Alberta | 158 | 11.8 |
| British Columbia | 165 | 12.3 |
| Manitoba | 49 | 3.7 |
| New Brunswick | 26 | 1.9 |
| Newfoundland and Labrador | 18 | 1.3 |
| Nova Scotia | 36 | 2.7 |
| Northwest Territories | 1 | 0.1 |
| Ontario | 523 | 39.0 |
| Prince Edward Island | 5 | 0.4 |
| Quebec | 318 | 23.7 |
| Saskatchewan | 42 | 3.1 |
| Yukon | 0 | 0.0 |
| Nunavut | 0 | 0.0 |
| Child Language | | |
| English | 1011 | 75.4 |
| French | 280 | 20.9 |
| Other | 49 | 3.7 |
| Prefer not to say | 1 | 0.1 |
| Food App on Child’s Phone^3^ | | |
| Yes | 870 | 64.9 |
| No | 454 | 33.9 |
| Prefer not to say | 17 | 1.3 |
| Weekday Screen Time | | |
| None | 13 | 1.0 |
| Up to 15mins | 28 | 2.1 |
| Up to 30 mins | 97 | 7.2 |
| Up to 1hr | 256 | 19.1 |
| Up to 2 hrs | 398 | 29.7 |
| Up to 3 hrs | 223 | 16.6 |
| Up to 4 hrs | 107 | 8.0 |
| More than 4hrs | 182 | 13.6 |
| Prefer not to say | 37 | 2.8 |
| Weekend Screen Time | | |
| None | 11 | 0.8 |
| Up to 15mins | 22 | 1.6 |
| Up to 30 mins | 65 | 4.8 |
| Up to 1hr | 170 | 12.7 |
| Up to 2hrs | 270 | 20.1 |
| Up to 3 hrs | 261 | 19.5 |
| Up to 4 hrs | 196 | 14.6 |
| More than 4 hrs | 303 | 22.6 |
| Prefer not to say | 43 | 3.2 |

*^1^Ethnicity was categorized as “majority” (i.e., only “White (European descent)” was selected) and “minority” (i.e., any other ethnicity group(s) were selected, including when in addition to “White (European descent)” being selected).^2^ Perceived income adequacy was categorized as “high” (Reponses of either very easy, easy, and neither easy nor difficult when asked how difficult or easy it is for you to make ends meet?) or “low” (responses of difficult or very difficult). ^3^Children were asked to select any of the food company mobile apps they had on their phones already, if any were selected, they were considered to have food apps on their phone.*
